# Surgical outcomes of transduodenal ampullectomy for early-stage ampullary carcinoma: a comparative analysis with pancreaticoduodenectomy

**DOI:** 10.1097/RC9.0000000000000544

**Published:** 2026-05-28

**Authors:** Anh The Pham, Cuong Manh Truong, Toan Quang Vu

**Affiliations:** a Department of Hepatobiliary and Pancreatic Surgery, Vietnam National Cancer Hospital, Hanoi, Vietnam; b Hanoi Medical University, Hanoi, Vietnam; c Department of Upper Gastrointestinal Medicine, Vietnam National Cancer Hospital, Hanoi, Vietnam

**Keywords:** ampullary carcinoma, pancreaticoduodenectomy, transduodenal ampullectomy

## Abstract

**Background/aims:**

Transduodenal ampullectomy (TDA) has been applied in selected cases of early ampullary carcinoma as a less invasive alternative to pancreaticoduodenectomy (PD). This study compared perioperative and oncologic outcomes between TDA and PD in patients with early-stage ampullary cancer.

**Methods:**

Patients who underwent curative-intent resection for ampullary carcinoma (Tis/high-grade dysplasia, T1, and T2) between August 2017 and February 2025 were retrospectively reviewed. Tumor staging was standardized according to the AJCC 8th edition. Clinicopathologic characteristics and surgical outcomes were analyzed between the TDA and PD groups.

**Results:**

Twenty-eight patients underwent TDA, and 35 underwent PD. The TDA group showed significantly shorter operative time, lower estimated blood loss, shorter postoperative hospital stay, and reduced overall complication rate. The incidence of clinically relevant postoperative pancreatic fistula was significantly lower after TDA. Lymph node metastasis was identified in 20.0% of the TDA group and 21.2% of the PD group. Five-year disease-free survival rates were comparable between the two groups in the T1 subgroup analysis.

**Conclusions:**

In carefully selected patients with Tis/HGD and T1 ampullary carcinoma, TDA combined with lymph node dissection provides favorable perioperative outcomes and comparable long-term disease control relative to PD. For T2 disease, PD remains the standard of care, while TDA may be considered only in highly selected cases unfit for radical surgery.

## Introduction

Carcinoma arising from the ampulla of Vater (AoV) accounts for a small proportion of gastrointestinal malignancies, representing less than 1% of cases, and is the second most common tumor within the periampullary region after pancreatic cancer^[^1,2^]^. Owing to its low incidence, evidence guiding management strategies is largely derived from retrospective series, and consensus recommendations remain limited.

Pancreaticoduodenectomy (PD), including pylorus-preserving pancreaticoduodenectomy (PPPD), has been regarded as the standard surgical treatment for resectable ampullary carcinoma because it allows *en bloc* tumor removal with systematic lymph node dissection (LND). Nevertheless, PD is a technically demanding procedure associated with substantial morbidity, such as postoperative pancreatic fistula, delayed gastric emptying, postoperative bleeding, and long-term endocrine insufficiency^[^3^]^.

HIGHLIGHTS

- Transduodenal ampullectomy (TDA) offers superior perioperative safety compared to pancreaticoduodenectomy (PD) for early-stage ampullary carcinoma.
- TDA significantly reduces operative time, intraoperative blood loss, and the duration of postoperative hospital stay.
- The incidence of clinically relevant postoperative pancreatic fistula is significantly lower following TDA than after PD.
- TDA combined with lymph node dissection achieves comparable 5-year disease-free survival to PD in carefully selected Tis and T1 cases.
- TDA represents a viable, less invasive alternative to PD for early-stage (Tis/T1) ampullary tumors, particularly in high-surgical-risk patients.

To reduce surgical burden, transduodenal ampullectomy (TDA) has been proposed for selected patients with early-stage disease confined to the ampulla or sphincter complex, particularly carcinoma *in situ* and pT1 tumors. The principal concern regarding TDA relates to oncologic adequacy, as margin status and lymph node metastasis are well-established determinants of recurrence and survival^[^3–5^]^. Previous investigations have explored predictive factors for nodal involvement, patterns of lymphatic dissemination, and recurrence characteristics to better define appropriate indications for limited resection^[^4–10^]^.

Despite these efforts, the oncologic role of TDA remains controversial. While several institutions have reported acceptable long-term outcomes with TDA combined with regional LND in early-stage AoV carcinoma^[^11–13^]^, the optimal extent of lymphadenectomy and patient selection criteria have not been clearly established. In addition, available studies are frequently limited by small cohorts or insufficient follow-up.

Therefore, the present study evaluated the surgical and oncologic outcomes of TDA performed at a single high-volume center and compared them with those of PD, with the aim of clarifying the feasibility of TDA as a treatment option for early AoV carcinoma.

## Materials and methods

### Patient selection and data collection

A retrospective review was performed using a prospectively maintained institutional database. Patients diagnosed with ampullary carcinoma who underwent TDA between August 2017 and February 2025 were included. During the same study period, patients treated with PD for ampullary neoplasms were identified for comparison.

Demographic variables and clinicopathologic data were collected, including age, sex, tumor size, pathologic T category, lymph node status, and tumor differentiation. Perioperative parameters and long-term oncologic outcomes were also analyzed.

Tumor staging was determined according to the 8th edition of the American Joint Committee on Cancer (AJCC) classification system. In line with the current World Health Organization criteria, low-grade dysplasia was categorized as benign, whereas high-grade dysplasia was classified as carcinoma *in situ* (Tis). Functional status was assessed using the Eastern Cooperative Oncology Group (ECOG) performance status and the American Society of Anesthesiologists (ASA) physical status score.

Postoperative complications were assessed using the Clavien–Dindo grading system^[^14^]^, with grade III or higher considered major morbidity. Clinically relevant postoperative pancreatic fistula and delayed gastric emptying were defined and graded according to the criteria established by the International Study Group of Pancreatic Surgery^[^15,16^]^.

### Indication and procedure for TDA

#### Indications

TDA was considered in the following clinical settings:

- Endoscopic papillectomy (EP) was deemed unsuitable, particularly in cases with extension into the distal bile duct or pancreatic duct.
- Incomplete resection or local recurrence following prior EP.
- Patients considered high risk for PD because of advanced age or significant comorbidities, including poorly controlled hypertension, diabetes mellitus, bronchial asthma, or cardiovascular disease, or those who declined PD after counseling.
- Preoperative diagnosis of ampullary adenocarcinoma staged as cTis–T1N0M0 according to the 8th edition of the AJCC TNM classification (2017), based on cross-sectional imaging (computed tomography or magnetic resonance imaging) and/or endoscopic ultrasonography.
- The institutional decision-making algorithm for the surgical approach was as follows: PD was the preferred treatment for fit patients with suspected T2 or higher-stage disease. TDA was considered for: (1) biopsy-proven Tis or EUS-staged T1N0 disease; (2) patients with significant comorbidities (ASA ≥ 3 or ECOG ≥ 2), making them high-risk for PD; or (3) patients who explicitly refused PD after thorough counseling.

#### Surgical procedure

**Figure 1.**
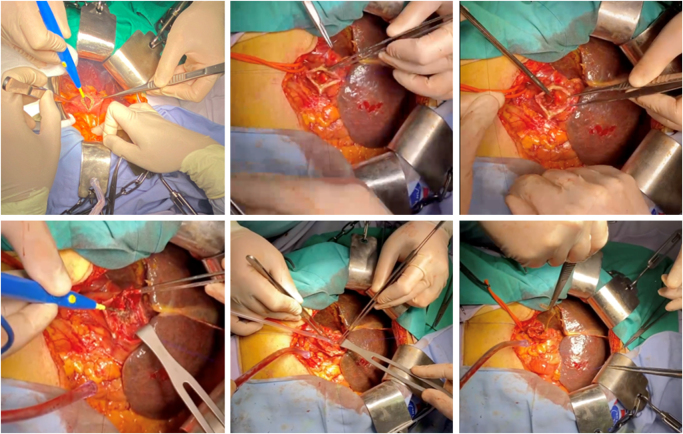
Operative technique: (1) After performing the Kocher maneuver, make a transverse incision at D2 over the lesion. (2) Suspend the duodenal wall for better exposure. (3) Use sutures to suspend the ampullary lesion. (4) Excise the lesion, including part of the duodenum, the distal common bile duct, and the pancreatic head. (5) Insert a gastric tube for bile duct dilation to ensure there is no stenosis. (6) Place a decompressive nasogastric tube into the D2 duodenum. (7) Perform lymph node dissection (Supplemental Digital Content Video 4, available at: http://links.Lww.Com/IJSCR/A56).

Surgical procedure is as follows (Fig. 1; Supplemental Digital Content Video 1, available at: http://links.lww.com/IJSCR/A53; Supplemental Digital Content Video 2, available at: http://links.lww.com/IJSCR/A54; Supplemental Digital Content Video 3, available at: http://links.lww.com/IJSCR/A55; Supplemental Digital Content Video 4, available at: http://links.lww.com/IJSCR/A56):

- Under general anesthesia, patients were placed in the supine position. A midline laparotomy (approximately 20 cm) was performed. The abdominal cavity was systematically explored to exclude peritoneal dissemination, distant metastases, or suspicious lymphadenopathy.
- Cholecystectomy was routinely carried out, followed by a Kocher maneuver to mobilize the duodenum and pancreatic head. A longitudinal duodenotomy was created on the second portion of the duodenum to expose the ampullary complex.
- The ampulla was excised *en bloc*, including a cuff of the surrounding duodenal wall, the distal common bile duct, and adjacent pancreatic tissue as required to achieve adequate margins. Intraoperative frozen-section analysis was performed to confirm negative resection margins.
- Reconstruction consisted of separate mucosa-to-mucosa anastomoses of the bile duct and pancreatic duct to the duodenal wall using absorbable sutures (4-0). Care was taken to avoid narrowing of the pancreatic duct during suturing. The duodenotomy was then closed in a continuous fashion.
- Regional LND was selectively performed in cases with suspected invasive carcinoma (cT1 stage on preoperative imaging or suspicious findings on intraoperative frozen section), including nodal stations 7, 8, 12, and 13.
- To ensure ductal patency, a tube was temporarily inserted to confirm an adequate bile duct lumen. An additional nasogastric tube was advanced into the duodenum for decompression. Two closed-suction drains were placed in the subhepatic space prior to abdominal closure.

### Statistical analyses

Categorical variables were expressed as numbers with corresponding percentages and were compared using the chi-square test or Fisher’s exact test, as appropriate. Continuous variables were presented as mean ± standard deviation or median with range, depending on data distribution. Between-group comparisons were performed using the Student’s *t*-test for normally distributed variables and the Mann–Whitney U test for nonparametric data.

Factors associated with tumor recurrence were evaluated using the Cox proportional hazards regression model. Variables assessed in univariate analysis included age, sex, tumor size, surgical procedure, intraoperative transfusion, pathologic T category, lymph node involvement, lymphovascular invasion, perineural invasion, and administration of adjuvant therapy. Parameters demonstrating statistical significance in univariate testing (*P* < 0.05) were subsequently entered into multivariate analysis.

Disease-free survival (DFS) was defined as the interval from the date of surgery to the detection of locoregional or distant recurrence. Patients without recurrence were censored at the time of the last follow-up or death unrelated to disease recurrence. Overall survival (OS) was calculated from the date of operation to death from any cause, with surviving patients censored at their most recent follow-up visit.

Survival curves were generated using the Kaplan–Meier method and compared using the log-rank test. All statistical analyses were conducted using SPSS version 25.0 (IBM Corp., Chicago, IL, USA). A two-sided *P*-value of < 0.05 was considered statistically significant.

This cohort study has been reported in line with the PROCESS 2025 guideline^[^17^]^.

## Results

Between August 2017 and February 2025, a total of 28 patients underwent TDA for ampullary carcinoma at our institution. During the same period, patients who received PD were screened for eligibility. Cases were excluded if they had non-ampullary malignancies, previous TDA, distant metastasis (M1), neoadjuvant chemotherapy, synchronous pancreatic cancer, benign pathology including adenoma or low-grade dysplasia, advanced pathologic stage (T3–T4), neuroendocrine tumor, or lymphoma. After applying these criteria, 35 patients were included in the PD group for comparative analysis. Within the PD cohort, three patients (8.6%) had pathologic T1 tumors, and 32 patients (91.4%) had T2 tumors. No cases of high-grade dysplasia or carcinoma *in situ* were identified in this group.

### Clinicopathologic characteristics of patients with malignant ampullary tumors

Baseline clinicopathologic features of patients with pHGD/Tis, T1, and T2 ampullary carcinoma are summarized in Table 1. Demographic variables, including age and ASA score, were comparable between the two groups. Preoperative tumor marker levels and tumor size did not differ significantly. Baseline ECOG performance status was comparable between groups (*P* > 0.05), although the PD group had a higher proportion of T2 tumors (91.4% vs. 10.7%, *P* < 0.001).

**Table 1:** Comparison of clinicopathologic findings between the transduodenal ampullectomy (TDA) and pancreaticoduodenectomy (PD) groups for malignant ampullary tumors (pHGD/Tis, T1, and T2).

Characteristic	TDA (*n* = 28)		PD (*n* = 35)		P*
Age (years), mean ± SD	62.8 ± 9.1		60.6 ± 12.6		0.144
Sex, *n* (%)					**0.003**
Male	21	(75.0%)	13	(27.1%)	
Female	7	(25.0%)	22	(62.9%)	
ECOG performance status, *n* (%)					0.652
0	18	(64.3%)	24	68.6%	
1	8	(28.6%)	9	25.7%	
2	2	7.1%	2	5.7%	
ASA score, *n* (%)					0.819
1	16	(57.1%)	21	(60.0%)	
2	12	(42.9%)	14	(40.0%)	
Preoperative endoscopic papillectomy, *n* (%)					0.076
Yes	7	(25.0%)	3	(8.6%)	
No	21	(75.0%)	32	(91.4%)	
Preoperative tumor marker, median (range)					
CA 19-9 (U/ml)	173.8	(0.6–1200.0)	16.6	(0.1–1270.0)	0.245
CEA (ng/ml)	4.8	(1.6–14.3)	41.3	(0.8–9490.0)	0.373
Tumor size (mm), mean ± SD	17.3 ± 4.6		20.7 ± 9.7		0.226
T stage (AJCC 8th edition)					**<0.001**
pHGD/Tis	10	(35.7%)	0	(0%)	
pT1	15	(43.6%)	3	(8.6%)	
pT2	3	(10.7%)	32	(91.4%)	
N stage (AJCC 8th edition)					0.473
pNx	0	(0%)	0	(0.0%)	
pN0	25	(89.3%)	28	(80.0%)	
pN1	3	(10.7%)	5	(14.3%)	
pN2	0	(0%)	2	(5.7%)	
Stage (AJCC 8th edition)					**<0.001**
0	9	(32.1%)	0	(0%)	
I	10	(35.7%)	0	(0%)	
IB	6	(21.4%)	13	(37.1)	
IIA	0	(0%)	10	(28.6%)	
IIB	0	(0%)	5	(14.3%)	
IIIA	3	(10.7%)	5	(3.0%)	
IIIB	0	(0%)	2	(5.7)	
Differentiation					0.351
Well	6	(21.4%)	2	(5.7%)	
Moderate	15	(53.6%)	23	(65.7%)	
Poor	3	(10.7%)	4	(11.4%)	
NA	4	(14.3%)	6	(17.1%)	
Lymphovascular invasion					**0.027**
Yes	5	(17.9%)	17	(48.6%)	
No	13	(46.4%)	9	(25.7%)	
NA	10	(35.7%)	9	(25.7%)	
Perineural invasion					**0.005**
Yes	2	(7.1%)	12	(34.3%)	
No	17	(60.7%)	15	(42.9%)	
NA	9	(32.1%)	8	(22.9%)	

ASA, American Society of Anesthesiologists; HGD, high-grade dysplasia; NA, not available; SD, standard deviation; Tis, carcinoma *in situ*. Bold *P*-values represent statistically significant differences between the TDA and PD groups in sex distribution, pathological T stage, AJCC stage, lymphovascular invasion, and perineural invasion.

^*^ *P*-values are from the Student’s *t*-test or Mann–Whitney test for continuous factors, and v^2^ (Fisher’s exact) test for categorical factors.

A higher proportion of female patients was observed in the PD group (*P* < 0.001). Regarding pathologic stage distribution, early-stage tumors (T1) were more frequently treated with TDA, whereas T2 lesions predominated in the PD group (*P* < 0.001).

LND was performed in 15 patients (53.6%) undergoing TDA, among whom three (20.0%) had nodal metastasis. In the PD group, lymph node involvement was identified in seven of 33 evaluable patients (21.2%). Tumor differentiation was similar between groups. However, lymphovascular invasion and perineural invasion were more frequently detected in patients who underwent PD (48.6% vs. 17.9%, *P* = 0.027; 34.3% vs. 7.1%, *P* = 0.005, respectively).

### Perioperative outcomes

Perioperative outcomes are presented in Table 2. Patients in the TDA group had significantly shorter operative times and a reduced length of postoperative hospitalization compared with those undergoing PD. Intraoperative blood loss was also significantly lower in the TDA group.

**Table 2:** Surgical and oncologic outcomes after transduodenal ampullectomy (TDA) and pancreaticoduodenectomy (PD) (pHGD/Tis, T1, and T2).

Outcomes	TDA (*n* = 28)		PD (*n* = 35)		P*
Operation time (min), mean ± SD	118.6 ± 36.8		246.5 ± 80.6		**<0.001**
Estimated blood loss (ml), median (range)	60	(0–700)	300	(0–1200)	**<0.001**
Intraoperative transfusion, *n* (%)					0.214
Yes	1	(3.6%)	5	(14.3%)	
No	27	(96.4%)	30	(85.7%)	
Hospital stays (days), median (range)	9	(6–22)	14	(7–33)	**<0.001**
Overall complications, *n* (%)	4	(14.3%)	16	(45.7%)	**0.008**
Major complications (CD grade ≥ III), *n* (%)	2	(7.1%)	4	(11.4%)	0.684
Clinically relevant POPF (Grade B/C), *n* (%)	5	(17.9%)	35	(100%)	**0.017**
Grade A	5	(17.9%)	28	(80.0%)	
Grade B	0	(0.0%)	7	(20.0%)	
Grade C	0	(0.0%)	0	(0.0%)	
DGE (grade ≥ B), *n* (%)	1	(3.6%)	5	(14.3%)	0.214
Duodenal stricture, *n* (%)	0	(0.0%)	–		
Complicated fluid collection, *n* (%)	2	(7.1%)	2	(5.7%)	0.448
Bleeding, *n* (%)	2	(7.1%)	3	(8.6%)	1.000
Readmission related with complications (<90 days)					1.000
Yes	1	(3.6%)	2	(5.7%)	
No	27	(96.4%)	33	(94.3%)	
Surgical mortality, *n* (%)	0	(0.0%)	0	(0.0%)	1.000
Recurrence, *n* (%)					0.973
No	17	(60.7%)	21	(60.0%)	
Yes	5	(17.9%)	7	(20.0%)	
NA	6	(21.4%)	7	(20.0%)	
Local	1	(20.0%)	2	(28.6%)	
Systemic	4	(80.0%)	3	(42.8%)	
Both	0	(0.0%)	2	(28.6%)	
Adjuvant therapy					0.371
No	1	(3.6%)	4	(11.4%)	
Yes	27	(96.4%)	31	(88.6%)	

CD, Clavien–Dindo classification; DGE, delayed gastric emptying; POPF, postoperative pancreatic fistula; SD, standard deviation. Bold values denote statistically significant differences in surgical and oncologic outcomes between the two groups (*P* < 0.05).

^*^ *P*-values are from the Student’s *t*-test or Mann–Whitney test for continuous factors, and v^2^ (Fisher’s exact) test for categorical factors.

Only one patient in the TDA cohort required an intraoperative transfusion, whereas five patients (14.3%) in the PD group received blood transfusions. The overall morbidity rate was significantly higher after PD (45.7% vs. 14.3%, *P* = 0.008). Although major complications occurred more frequently in the PD group (11.4% vs. 7.1%), this difference did not reach statistical significance. There was no 90-day postoperative mortality in either group.

### Long-term outcomes

The median duration of follow-up was 23 months (range: 1–73 months) in the TDA group and 28 months (range: 6–72 months) in the PD group. Patterns and frequency of tumor recurrence were comparable between the two surgical approaches. The proportion of patients receiving adjuvant therapy did not differ significantly (Table 2).

**Figure 2.**
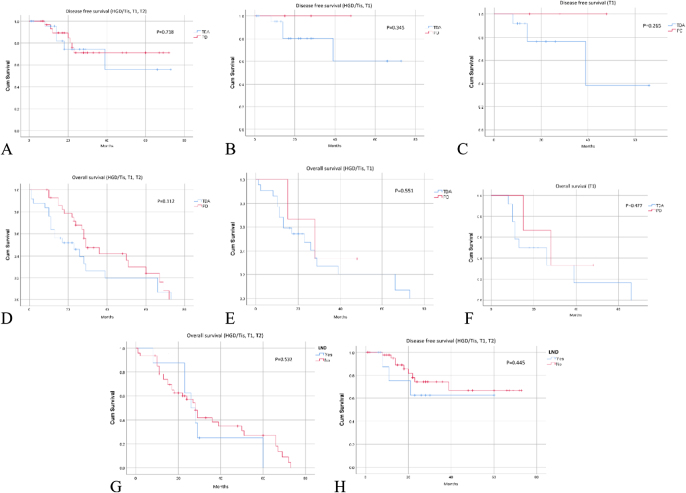
The survival outcomes of patients with malignant ampullary tumors after undergoing TDA or PD were examined. There was no significant difference in the 5-year disease-free survival (DFS) between the TDA and PD groups, regardless of the inclusion of T2 cases (a, b). Similarly, the overall survival (OS) rates were comparable between the two groups, irrespective of T2 cases (d, e). For the T1 population alone, the 5-year DFS and OS rates did not differ significantly between the TDA and PD groups (c, f). Additionally, when analyzing the TDA group based on whether lymph node dissection (LND) was performed, LND did not impact the 5-year DFS (g) or OS (h).

Kaplan–Meier analysis demonstrated no significant difference in 5-year DFS between the TDA and PD groups. This finding remained consistent when patients with T2 tumors were included in the analysis (Fig. 2a, b).

OS analysis similarly revealed no statistically significant difference between the two groups, irrespective of T2 inclusion (Fig. 2d, e). Among patients with T1 disease alone, both 5-year DFS and OS were comparable between TDA and PD (Fig. 2c, f).

Within the TDA cohort, additional subgroup analysis was conducted based on whether LND had been performed. The presence or absence of LND did not significantly influence 5-year DFS or OS (Fig. 2g, h).

Detailed clinicopathologic features and outcomes of the three patients with T2 tumors treated by TDA are presented in Table 3. Two patients remained alive without evidence of recurrence at the last follow-up. One patient developed locoregional recurrence 18 months after surgery and declined further treatment, including completion PD or adjuvant chemotherapy; this patient died 6 months after recurrence due to disease progression.

**Table 3:** Clinicopathologic findings of T2 patients after TDA.

No	Age/sex	Comorbidity	N stage	Histologic differentiation	LVI/PNI	Adj.Tx	Recurrence site	Time to recur (months)	Treatment for recurrence	Follow-up period(months)	Disease-related death
1	57/M	-	N0	Well	No	Gem/Cis	-	-	-	11	No
2	62/M	Hypertension	N0	Moderate	Yes	Gem/Cis	-	-	-	1	No
3	62/F	-	N0	Well	UK	Gem/Cis	Local	18	Palliative care	-	24

Adj.Tx, adjuvant treatment; Cis, cisplatin; Gem, gemcitabine; LVI/PNI, lymphovascular invasion/perineural invasion; UK, unknown.

At the 5-year follow-up mark, five patients in the TDA group and eight patients in the PD group remained at risk. Kaplan–Meier survival estimates at 5 years should be interpreted with caution due to the limited number of patients reaching this time point.

Subgroup analysis of T1 patients showed no significant difference in 5-year DFS (*P* = 0.82) or OS (*P* = 0.75) between TDA and PD.

### Risk factors for tumor recurrence

The results of univariate and multivariate Cox regression analyses evaluating factors associated with recurrence are summarized in Table 4. The surgical approach (TDA vs. PD) was not significantly associated with recurrence risk (*P* = 0.719).

**Table 4:** Univariate and multivariate analyses of risk factors affecting recurrence in patients with malignant ampullary tumors (≥ pHGD/Tis) undergoing transduodenal ampullectomy (TDA) or pancreaticoduodenectomy (PD).

Variable	No	Univariate			Multivariate		
		HR	95% CI	P*	Exp(B)	95% CI	P*
Age, years							
≤ 60	25						
> 60	35	0.441	0.119–1.635	0.221			
Sex							
Male	29						
Female	28	1.226	0.395–3.811	0.724			
Tumor size							
< 18 mm	18						
≥ 18 mm	39	1.710	0.502–5.831	0.391			
Type of resection							
PD	32						
TDA	25	1.235	0.390–3.906	0.719			
Intraoperative transfusion							
No	53						
Yes	4	1.156	0.149–8.962	0.890			
T classification							
Tis/T1	25						
pT2	32	0.899	0.238–3.400	0.876			
LN metastasis							
No	47						
Yes	10	1.656	0.445–6.161	0.452			
Differentiation							
Well	8						
Moderate/poor	49	1.026	0.224–4.687	0.974			
Lymphovascular invasion							
No	21						
Yes	20	0.291	0.060–1.407	0.125			
Perineural invasion							
No	30						
Yes	14	2.044	0.456–9.162	0.350	0.096	0.010–0.931	**0.043**
Adjuvant therapy							
No	4						
Yes	40	0.040	0.000–606.610	0.512			

AJCC, American Joint Committee on Cancer; CI, confidence interval; HR, hazard ratio; LN, lymph node; PD, pancreaticoduodenectomy; TDA, transduodenal ampullectomy. Bold values represent statistically significant results showing that perineural invasion was an independent risk factor for recurrence in the multivariate Cox proportional hazards analysis (*P* < 0.05).

* *P*-values are from the Cox proportional hazard model. Bold *P*-values indicate statistical significance (*P* < 0.05). Total number of recurrence events = 12. Due to the limited number of events, themultivariate model was restricted to variables with *P* < 0.1 in univariate analysis or clinically significant factors to avoid overfitting.

On univariate analysis, none of the evaluated clinicopathologic variables – including age, sex, tumor size, intraoperative transfusion, pathologic T category, lymph node metastasis, tumor differentiation (moderate/poor), lymphovascular invasion, perineural invasion, and receipt of adjuvant therapy – demonstrated a statistically significant relationship with recurrence (all *P* > 0.05). These findings may be influenced by the relatively small cohort size and the limited duration of follow-up.

In the multivariate model, perineural invasion was identified as an independent prognostic factor for recurrence (*P* = 0.043). Other variables, including intraoperative transfusion, T classification, nodal status, tumor differentiation, lymphovascular invasion, and adjuvant therapy, did not retain statistical significance.

## Discussion

The optimal surgical strategy for early ampullary carcinoma remains controversial. TDA offers a limited resection approach, whereas PD provides radical *en bloc* removal with systematic lymphadenectomy. Since its initial description by Halsted in the late 19th century and the subsequent standardization of PD by Whipple, the balance between oncologic radicality and surgical morbidity has continued to shape treatment decisions^[^13,18–21^]^.

PD has long been regarded as the standard treatment for resectable ampullary cancer because of its ability to address potential lymphatic spread. However, the procedure is associated with considerable postoperative morbidity, reported in 33%–52% of cases^[^21^]^. In the present study, overall complication rates were significantly higher following PD, consistent with previous reports. These findings support consideration of limited resection in selected patients, particularly those with substantial comorbidities.

Several comparative studies have evaluated the oncologic adequacy of TDA. A large Korean multicenter analysis, including 486 patients with pTis–T2 ampullary carcinoma, reported comparable 5-year disease-free and OS between PPPD and TDA when all early stages were considered collectively^[^21^]^. Notably, the PPPD group demonstrated more advanced tumor characteristics, including larger tumors, higher T category, and more frequent lymphovascular invasion. Although survival was reportedly superior in PPPD among T1 patients in that study, LND did not significantly influence survival within the TDA cohort. The authors ultimately recommended PPPD as the standard surgical approach.

Other single-institution studies have suggested that TDA with routine LND may achieve acceptable oncologic outcomes in carefully selected patients^[^13,22^]^. In our series, LND was performed in approximately half of the TDA cases, with a nodal metastasis rate comparable to that observed in the PD group. Recurrence patterns and 5-year survival outcomes were similar between surgical approaches, including analyses incorporating T2 tumors. These findings suggest that limited resection may provide adequate disease control in selected early-stage cases, although interpretation must consider potential selection bias.

The adequacy of lymphadenectomy remains an unresolved issue. Some investigators advocate intraoperative frozen-section assessment of lymph nodes and margins to guide the decision to proceed with TDA or convert to PD^[^13,18^]^. Such an approach may optimize oncologic safety; however, logistical constraints may limit its routine application. In our cohort, the absence of standardized intraoperative nodal assessment represents a limitation.

A recent systematic review and meta-analysis comparing endoscopic ampullectomy, surgical ampullectomy (including TDA), and PD demonstrated higher R0 resection rates with surgical approaches compared with endoscopic treatment, while PD was associated with greater morbidity^[^23^]^. Recurrence rates did not significantly differ among treatment modalities. These pooled data highlight the trade-off between surgical radicality and perioperative risk and underscore the importance of patient selection. Technically, TDA involves duodenotomy with careful identification of the bile and pancreatic ducts, circumferential excision of the ampullary complex with adequate margins, and reconstruction through duct-to-mucosa anastomosis to the duodenal wall^[^18,19^]^. Intraoperative frozen-section analysis of resection margins is essential to ensure complete tumor removal and to determine whether conversion to PD is warranted. Furthermore, as demonstrated in other rare surgical pathologic entities such as adult colonic intussusception secondary to lipoma, a meticulous clinicopathologic correlation is paramount in guiding the appropriate extent of surgical intervention and ensuring optimal patient outcomes^[^24^]^.

Taken together, our findings align with prior reports suggesting that TDA may represent a reasonable alternative to PD in selected patients with early-stage ampullary carcinoma. Nevertheless, the higher prevalence of adverse pathologic features in the PD group and the relatively limited follow-up period in our study warrant cautious interpretation.

Several limitations of this study must be acknowledged. First, the inherent baseline imbalance in T-stage between the TDA (predominantly Tis/T1) and PD (predominantly T2) groups introduces selection bias, reflecting the clinical reality where PD is reserved for more advanced lesions. Second, the median follow-up of 23‒28 months is relatively short for definitive 5-year survival claims; thus, long-term oncologic outcomes require validation in larger cohorts with longer follow-up. Third, the potential for under-staging in the TDA group exists due to less extensive lymphadenectomy compared to the standard PD. Finally, the inclusion of three T2 patients in the TDA arm represents “compromise” procedures for medically unfit patients and should not be interpreted as a recommendation for TDA in T2 disease.

## Conclusion

PD remains the reference surgical procedure for resectable ampullary carcinoma. However, TDA offers a safe and oncologically acceptable alternative in carefully selected patients with early-stage (Tis/T1) disease, particularly those with substantial comorbidities. Accurate preoperative staging and intraoperative margin confirmation are mandatory to ensure oncologic safety.

## Data Availability

The datasets generated and analyzed during the current study are available from the corresponding author on reasonable request.

## Research registration unique identifying number (UIN)

This cohort study has been reported in line with the PROCESS 2025 guidelines.

Our procedures adhered to the Declaration of Helsinki. This article was registered in “ResearchRegistry.com” with the identifying number being “researchregistry11818”.
